# Impacts of Rapid Desiccation on Oxidative Status, Ultrastructure and Physiological Functions of *Syzygium maire* (Myrtaceae) Zygotic Embryos in Preparation for Cryopreservation

**DOI:** 10.3390/plants11081056

**Published:** 2022-04-13

**Authors:** Karin van der Walt, David J. Burritt, Jayanthi Nadarajan

**Affiliations:** 1 Ōtari Native Botanic Garden, Wellington City Council, 150 Wilton Road, Wellington 6012, New Zealand; 2 School of Agriculture and Environment, Massey University, Palmerston North 4410, New Zealand; 3 Department of Botany, University of Otago, Dunedin 9016, New Zealand; david.burritt@otago.ac.nz; 4 Fitzherbert Science Centre, The New Zealand Institute for Plant and Food Research Limited, Batchelar Road, Palmerston North 4474, New Zealand; jayanthi.nadarajan@plantandfood.co.nz

**Keywords:** critically endangered, differential scanning calorimetry, recalcitrant, ROS, TEM, thermal transitions, threatened

## Abstract

*Syzygium maire* is a highly threatened Myrtaceae tree species endemic to New Zealand. Due to its recalcitrant seed storage behaviour, cryopreservation is the only viable long-term ex situ conservation option for this species. This study investigated viability, oxidative stress, thermal properties, and ultrastructure of zygotic embryo axes (EAs) desiccated to various moisture contents (MC). Fresh EAs had a MC of c. 1.9 g/g with 100% viability but rapid desiccation to MC < 0.3 g/g significantly reduced viability and decreased the activities of the enzymatic antioxidants superoxide dismutase, catalase and glutathione peroxidase, with a sevenfold increase in the production of protein carbonyls and lipid peroxides. Differential Scanning Calorimetry analysis showed no thermal events in EAs desiccated to a MC of <0.2 g/g, indicating that all freezable water had been removed, but this was lethal to both EAs and enzymatic antioxidants. The ultrastructure of desiccated EAs showed signs of plasmolysis, while fully hydrated EAs exposed to cryogenic temperature had ultrastructural disintegration and membrane damage. The decline in enzymatic antioxidant activities and the increase in lipid peroxidation suggest that *S. maire* EA viability loss is due to oxidative stress rather than structural impacts.

## 1. Introduction

Strategies for the long-term storage of desiccation-sensitive germplasm remain a challenge for many commercially important crops and endangered wild plant species [1]. Although cryopreservation provides a viable strategy for the long-term conservation of desiccation-sensitive species, its application is challenged because recalcitrant seeds are generally large, shed at high moisture contents, and are metabolically active [2]. One way to decrease the size of the material used is through the surgical removal of zygotic embryos from recalcitrant seeds [3,4,5,6]. 

For cryopreservation to be successful, it is essential that most of the freezable water from the plant tissue is removed to prevent the formation of lethal ice crystals [7]. The effects of partial dehydration on inducing oxidative stress in recalcitrant axes and zygotic embryos have been identified as a main source of cryoinjury in recalcitrant species [7,8,9]. When the metabolically active cells of recalcitrant species are dehydrated to intermediate moisture content (MC) levels, the cells continue to respire but can no longer effectively scavenge toxic metabolic by-products, including Reactive Oxygen Species (ROS) [10]. In plant cells under normal circumstances, ROS, e.g., superoxide (•O_2_), can be effectively removed by enzymatic antioxidants such as superoxide dismutase (SOD), catalase (CAT), glutathione peroxidase (GPOX) and other peroxidases, and non-enzymatic antioxidants such as ascorbate (AsA) and glutathione (GSH) [7,11,12]. However, under conditions resulting in ROS build-up, secondary oxidative reactions that result in cellular damage can occur. For example, ROS can attack proteins resulting in the formation of protein carbonyls and oxidative damage to lipid membranes that cause the formation of lipid peroxides [9]. Cell membranes have been identified as particularly susceptible to oxidative injury during metabolism disruption in recalcitrant seeds [2] and therefore, the accumulation of lipid peroxides is widely used as a biomarker of oxidative stress during desiccation of the seed of recalcitrant species [13,14]. The exogenous application of antioxidants, such as AsA and GSH, has successfully strengthened the ROS scavenging capacity of plants under abiotic stress conditions [15] and increased survival and regeneration following cryopreservation [16,17]. 

Despite the low, or lack of, tolerance to desiccation in recalcitrant species, it is essential to remove excess moisture from cells to prevent the formation of lethal ice crystals during freezing. Vitrification, when liquids are solidified without crystallization, has been achieved through the desiccation of plant material [18,19]. Following vitrification, the system is amorphous and comprises a glassy state that lacks an organized structure but has the mechanical and physical properties of a solid [20]. To evaluate the efficiency of desiccation to achieve a glassy state, thermal analysis using differential scanning calorimetry (DSC) can be used. DSC analyses water thermal properties of plant material by quantifying the amount of freezable water in the tissue and by confirming if a stable glassy state is achieved, which will prevent devitrification/recrystallization during the rewarming of the tissues (see [21] and references within). 

Lowering MC in recalcitrant tissues causes oxidative stress and the physical structure of their cells can also be compromised during water removal [22]. Ultrastructural studies can be used to compare subcellular organisation at various steps of the desiccation and freezing process to ensure cell integrity is maintained [23,24,25]. By comparing subcellular organisation between fresh, desiccated, and desiccated/frozen EAs, fatal changes in a subcellular organisation can be identified and provide essential information for developing species-specific cryopreservation protocols. 

Another aspect of the cryopreservation process that requires optimisation is the type of solution used for rehydration of the cryopreserved material prior to regeneration. Although rehydration of isolated axes is normally done through plunging into distilled water [1], the addition of Ca^2+^ and Mg^2+^ cations to the rehydration medium has been found to increase survival in Amaryllid species [26] and to improve regeneration in *Quercus robur* and *Trichilia dregeana* [27]. Rehydration solutions containing antioxidants such as ascorbic acid have also been found to increase survival in various species [28]. Like the steps in the cryopreservation process, rehydration techniques and solutions must be optimised for every species. 

**Figure 1:**
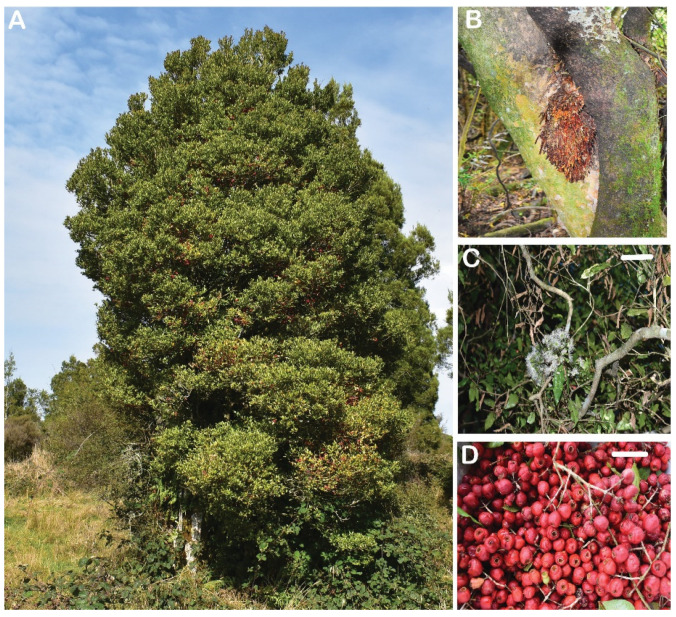
*Syzygium maire* in habitat (**A**) with pneumatophores on the main trunk (**B**). Creamy white flowers are present between February and April (**C**) with fruit development over 8–11 months, producing mature berries in autumn (**D**). Scale bar = 3 mm.

*Syzygium maire* (Myrtaceae) is a 16 m high evergreen tree endemic to New Zealand (Figure 1A). Pneumatophores, which are lateral roots adapted for oxygen uptake, enable it to grow in swamps, mangroves and muddy environments (Figure 1B). The creamy white flowers appear between January and April, with fleshy berries developing over 8–11 months (Figure 1C,D). Significant loss of habitat, coupled with a threat from the fungal pathogen myrtle rust (*Austropuccinia psidii*), has resulted in *S. maire* being listed as Nationally Critical in the New Zealand Threat Classification System [29]. Seeds and embryos of *S. maire* are recalcitrant and do not tolerate desiccation to MCs below 20% [30,31]. 

This study aimed to explain the observed effects of rapid desiccation on various indicators of oxidative stress as a function of MC, viability, and thermal transitions in *Syzygium maire*. The specific objectives of the study were to: (1) determine the impact of desiccation on embryo viability and plantlet development, (2) determine the suitability of evaporative desiccation in the cryopreservation of *S. maire*, (3) use antioxidant enzymes (SOD, CAT and GPOX) and oxidative damage markers (lipid peroxides and protein carbonyls) to quantify the oxidative stress induced during desiccation, (4) measure water thermal transitions using DSC in desiccated zygotic embryos to ensure the glassy state is achieved following rapid desiccation, and (5) investigate subcellular impacts of desiccation and freezing on zygotic embryos. 

## 2. Results

### 2.1. Embryo Response to Desiccation and Cryopreservation

**Figure 2:**
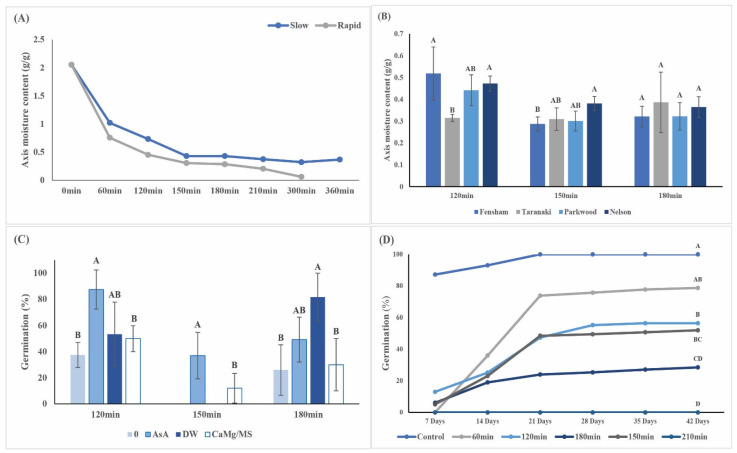
Comparative moisture loss from embryonic axes (EAs) was significantly faster during rapid desiccation (**A**). Each datum point is the mean of 40 individual EAs. Significant differences in moisture loss between seed lots were recorded during rapid desiccation for 120 min and 150 min, but moisture contents were similar after 180 min (**B**). Rehydration with ascorbic acid (AsA) improved embryo survival following rapid desiccation for 120 and 150 min (**C**). Rapid desiccation delayed the onset of radicle emergence (indicating survival) across all desiccation treatments, with final survival significantly lower for longer desiccation treatments (**D**). Letters above bars and line graphs for each desiccation treatment indicate significant differences at *p* < 0.05 based on Tukey’s HSD. Error bars represent the SD of the mean.

The MC of fresh EAs was similar across years and provenances and ranged from 1.66 ± 0.18 g/g to 1.93 ± 0.19 g/g. Typically, rapid desiccation reduced the MC to approximately 0.44 g/g within 120 min, while it took 150 min to achieve the same MC during slow drying (Figure 2A). Moisture loss during the first 60 min was rapid in both methods, but significantly more moisture was removed when using rapid desiccation (0.75 ± 0.13 g/g; *p* < 0.001) compared with slow desiccation (1.02 ± 0.25 g/g). Based on this, rapid desiccation was applied to all further desiccation experiments. MC levels for EAs from different seed lots were significantly different after 120 min and 150 min of rapid desiccation but after 180 min, MCs were similar across all seed lots (Figure 2B). Rehydration with AsA significantly improved embryo survival following 120- and 150 min desiccation, while improved survival was also achieved after rehydration with AsA and distilled water (DW) following 180 min desiccation (Figure 2C). Desiccation delayed the onset of radicle emergence by at least 7 days, while final embryo survival was also significantly affected by desiccation time (Figure 2D). At MC levels below 0.28 g/g, the development of plantlets was significantly reduced, with less than 50% of germinated EAs producing leaves (Supplementary Figures S1A,B). After four months in vitro, all surviving plantlets from non-desiccated and desiccated EAs were similar in size (14.1 ± 3.1 mm and 18.7 ± 7.6 mm, respectively; Supplementary Figure S1C), although out of all the treatments, three plantlets produced shoots with no roots (Supplementary Figure S1D). No plants survived cryopreservation (+LN), irrespective of the desiccation method or moisture content (Table 1).

### 2.2. Oxidative Stress

**Figure 3:**
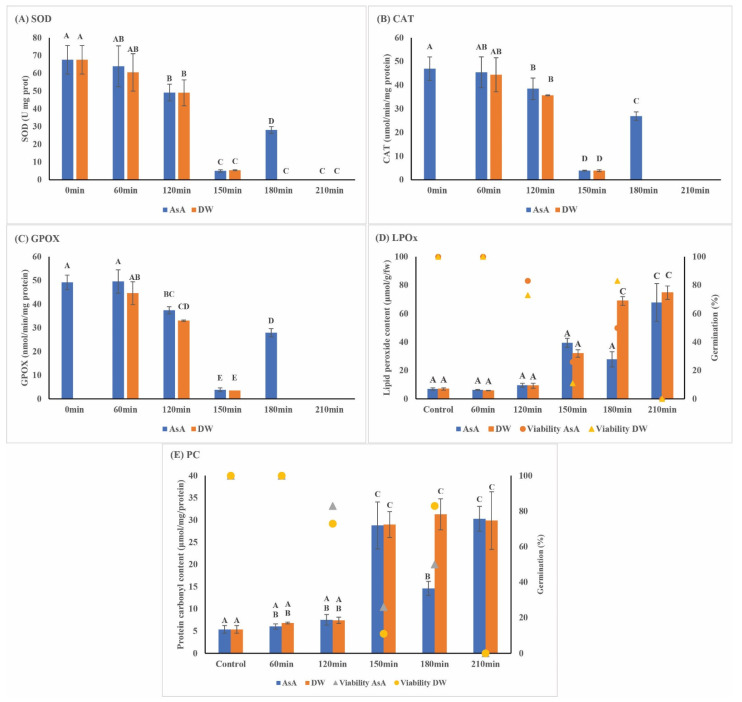
Superoxide dismutase (SOD) (**A**), catalase (CAT) (**B**) and glutathione peroxidase GPOX (**C**) activities in the embryonic axis (EA) of *Syzygium maire* at various desiccation treatments following rehydration in ascorbic acid (AsA) or distilled water (DW) for 30 min. The impact of desiccation on lipid peroxide content (LPOx) (**D**) and protein carbonyl (PC) content (**E**) of *Syzygium maire* EA. Symbols represent mean viability for each treatment, error bars represent SD of the mean. The letters above the bars indicate a significant difference based on Tukey’s HSD (*p* < 0.05).

In the present study, except for 180 min desiccation followed by rehydration with AsA, desiccation of EAs to MC below 0.3 g/g resulted in a ten-fold decrease in all three enzymatic antioxidants (SOD, CAT and GPOX) with a five-fold increase in PC and LPOx (Table 2). No enzymatic activity was detected at MC of <0.25 g/g, and this corresponded with a total loss of viability. There was a significant positive correlation between EA viability and SOD (*r* = 0.998), CAT (*r* = 0.78) and GPOX (*r* = 0.79; *p* = 0.01) activities. Desiccated *S. maire* EAs rehydrated in AsA had slightly higher SOD, CAT and GPOX activities compared with EAs rehydrated in DW, though this difference was not significant (Figure 3A–C). A significant accumulation of LPOx and protein carbonyl contents was associated with a decrease in MC (Figure 3D,E).

### 2.3. Differential Scanning Calorimetry (DSC)

**Figure 4:**
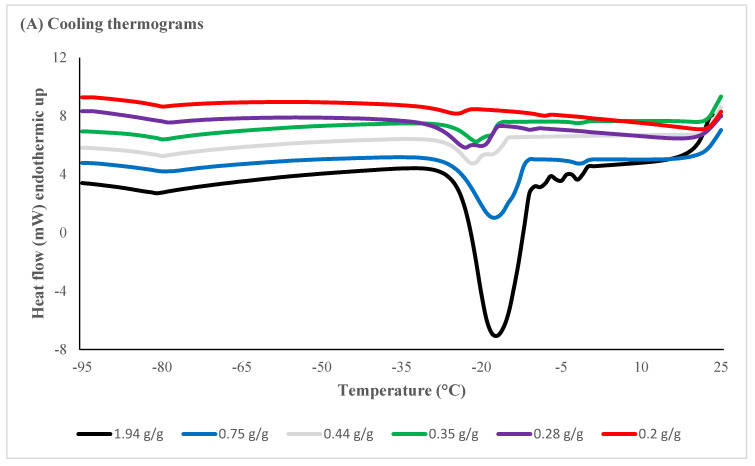

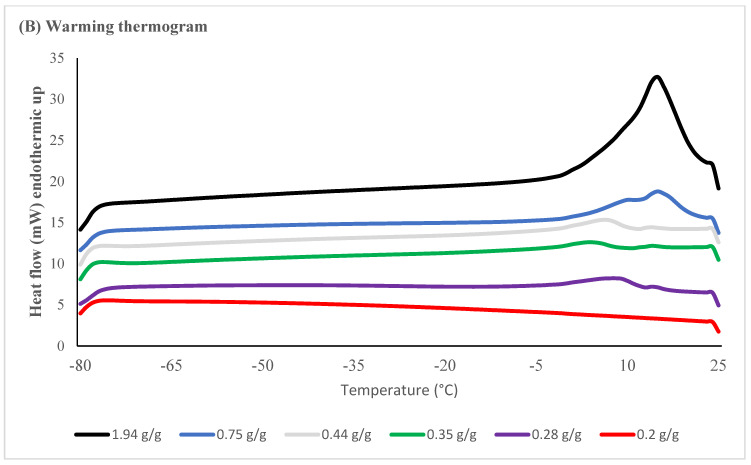
Differential Scanning Calorimetry warming (**A**) and cooling (**B**) thermograms for *Syzygium maire* embryonic axes (EAs) following rapid desiccation to various moisture contents (MC). The results are for a single EA, which was rapidly desiccated for 0 min (solid black line), 60 min (blue line), 120 min (solid light grey line), 150 min (green line), 180 min (purple line), or 210 min (red line) to achieve indicated MC levels.

The DSC thermograms provided measurements for water thermal phase transitions, including temperatures and enthalpies of water crystallization, ice melting and glass transition temperatures (Supplementary Data Table S1). The amount of osmotically active (freezable) water, quantified using the area under melting transition, declined in response to desiccation times (Table 3). Onset and end temperatures of ice nucleation and ice melt, as well as areas of crystallization/melt and enthalpies, are illustrated in Figure 4. The melt onset temperature was significantly lower in EAs desiccated to reach MC of <0.44 g/g (120 min or longer) but there was no significant difference in the melt onset temperature in samples desiccated for between 120 and 180 min. No thermal event was evident in EAs desiccated for 210 min (<0.20 g/g) (Table 3). Ice nucleation was inhibited after 210 min desiccation to reach a moisture content of c. 0.21 g/g with no thermal events recorded on rewarming (Figure 4A). Enthalpies of melt were reduced to smaller/insignificant thermal events recorded between 5 and 10 °C following desiccation of 120 min or longer (Figure 4B).

### 2.4. Ultrastructural Observations

**Figure 5:**
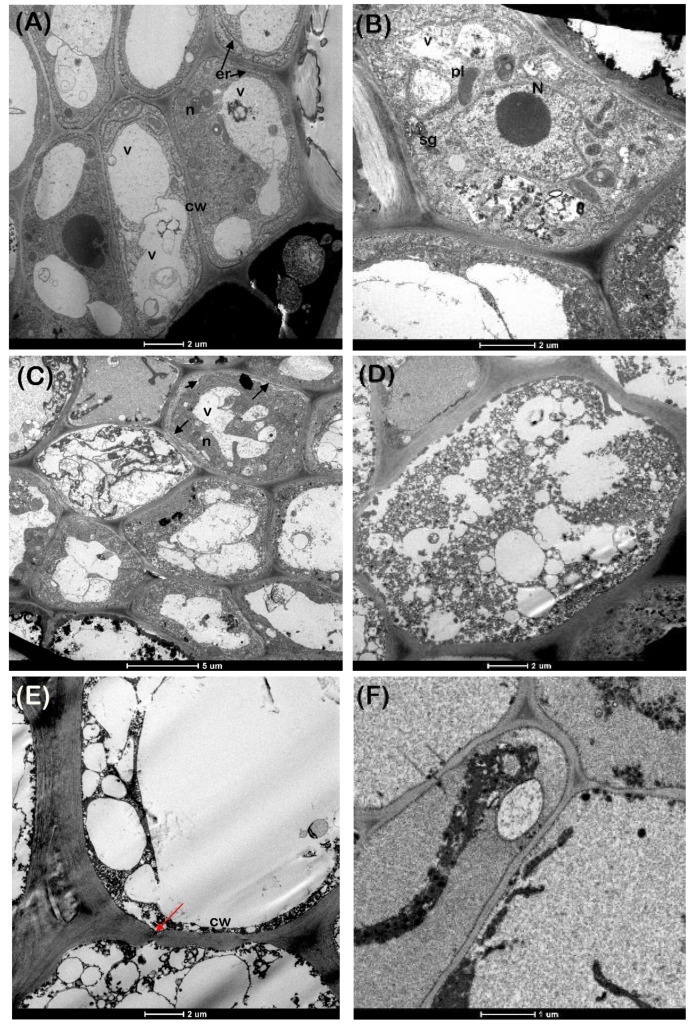
The ultrastructure of fresh *Syzygium maire* embryonic axes (EAs) typically had large vacuoles (V), irregularly shaped nuclei (N) and well-developed endoplasmic reticulum (er) (**A**). Good ultrastructural preservation was evident following desiccation to <0.3 g/g (**B**), although plasmolysis (arrows) was evident together with an increase in the vacuole (v) (**C**). Disintegration of cells (**D**), as well as damage to cell membranes (**E**), were evident following freezing of EAs with MC > 0.75 g/g, while cell membranes appeared intact after freezing of EAs desiccated to <0.3 g/g (**F**).

Meristematic cells of hydrated EAs, as illustrated in Figure 5A, were compact and characterised by large vacuoles. Mitochondria were frequent and had clearly defined cristae, with little polysome formation. Plastids contained well-defined starch grains and internal membranes were well developed. Cell walls were smooth and did not show any protrusions or disruptions with cells containing large nuclei. In all cells, the plasmalemma was closely appressed to the cell walls. Good ultrastructural preservation was still evident after desiccation to <0.3 g/g MC with regular-shaped nuclei and dense plastids, although an occasional abnormality in the form of plasmolysis was encountered with an increase in the number of vacuoles (Figure 5B,C). The overall deterioration of the ultrastructure (Figure 5D) was apparent when EAs with MC > 0.75 g/g were exposed to liquid nitrogen with cell wall disruption evident (Figure 5E), while cell membranes appeared intact following exposure to LN at MCs of <0.3 g/g (Figure 5F).

## 3. Discussion

Like many recalcitrant-seeded species, *S. maire* seeds are shed at high MCs with seeds and EAs remaining sensitive to desiccation [31]. It is now generally accepted that rapid drying of EAs permits survival to lower MC (reviewed by 10) due to less time available for aqueous-based deleterious reactions to occur [7,10,22]. The loss of enzymatic antioxidant capacity is often accompanied by a decline in viability following desiccation or cryopreservation [32]. CAT and GPOX are involved in the metabolism of hydrogen peroxide (H_2_O_2_), which is a product of SOD catalysed dismutation of •O_2_ [33]. Enzymatic antioxidant activities (SOD = 67.6; CAT = 49.6 and GPOX = 49.2) in *S. maire* EAs with high MCs (>0.75 g/g) were similar to those recorded in mature zygotic embryos of *Juglans regia* [34]. A decrease in embryo viability in *S. maire* was recorded at MCs of below 0.44 g/g, which was achieved after approximately 120 min of rapid desiccation. Further desiccation resulted in more survival loss accompanied by a significant drop in the activities of all three enzymes. This was, however, not unexpected—a drop in protective enzymes during the desiccation of recalcitrant EAs was previously reported for *Mudhuca latifolia* [35], *Quercus robur* [14,32], and *Trichillia dregeana* [36]. SOD, CAT and GPOX all continued to decline progressively with the desiccation of *S. maire* EAs; this was in contrast with studies on *T. dregeana* in which APX, which also metabolises H_2_O_2_, initially declined then increased in response to rapid desiccation [36]. The loss of SOD, CAT and GPOX activities could indicate that these enzymes were damaged because of high ROS levels and oxidative damage, and so are no longer able to effectively scavenge free radicals and/or the synthesis of new proteins was inhibited. The high levels of protein carbonyls observed as well as the decline in SOD, CAT and GPOX activities supports the view that oxidative damage to proteins including enzymes was occurring. 

Cell membranes comprise two components, a lipid bilayer and membrane proteins, and these membranes are usually the first target for stress during excessive ROS levels [15]. The ROS attack unsaturated fatty acids, which results in the peroxidation of membrane lipids and eventually leads to cell death [10]. This was evident in *S. maire* EAs in which the first significant increase in LPOx was at MC of 0.3 g/g with close to a 10-fold increase in LPOx following desiccation to less than 0.2 g/g MC. Interestingly, there was a negative correlation between the increase in LPOx and EA viability (*r* = −0.655), indicating that viability loss is likely not only due to membrane disintegration. 

Exogenous application of AsA to plants under abiotic stress has resulted in positive, negative and neutral responses (see [15] and references within). This study found that desiccated *S. maire* EAs rehydrated in AsA had slightly higher SOD, CAT and GPOX activities compared with EAs rehydrated in distilled water, though this difference was not significant. It is possible that 30 min rehydration in AsA did not allow sufficient time for cellular uptake, thereby minimizing the impact AsA had on antioxidant activities. Interestingly, in EAs desiccated to 0.28 g/g, SOD, CAT, and GPOX activity was significantly higher in EAs rehydrated with AsA compared with distilled water; it also had higher antioxidant activities compared with EAs desiccated to 0.3 g/g. The most likely explanation for this is that EAs used in the 0.28 g/g MC treatment were larger and therefore did not consistently reach low MCs, resulting in less oxidative stress. Unlike somatic embryos, zygotic embryos are not uniform in size and consist of heterogeneous tissues, which likely influences desiccation intensity and duration [22,37]. Embryo anatomy and tissue topography have also been implicated in low rates of seedling recovery or abnormal growth following desiccation and/or cryopreservation [1,26,38]. Despite these variances, MCs selected for desiccation and cryopreservation are based on embryo bulk water content, which is obtained from mass-weighted averages [6]. Similar challenges were found in the present study; for example, MCs between replicates following 150 min rapid desiccation ranged from 0.27 to 0.46 g/g and this was also reflected in viability tests where replicates from longer desiccation treatments sometimes had higher survival compared with shorter treatments (Figure 3C). It is postulated that not all EAs within the same desiccation treatment reached MCs based on bulk water contents, and larger embryos therefore showed higher antioxidant activity and viability. This was also evident in thermograms of individual EAs, which showed a ten-fold difference in the onset temperature of ice crystals between various EAs desiccated for 60 min (data not shown). 

The MC in biological material is a critical factor for successful cryopreservation of living tissue [39,40] with MCs of 0.40–0.25 g/g reported in the successful cryopreservation of recalcitrant embryos [1,11]. Thermal transitions of *S. maire* EAs were used to determine the amount of water available to freeze at various MC levels. In non-desiccated EAs, there was a large endothermic peak with an onset temperature close to 0 °C, indicating that this endothermic peak was due to ice formation during the cooling phase. The endothermic events in desiccated EAs became progressively smaller with EAs desiccated to <0.44 g/g, displaying melt enthalpies of 37.78 to 65.91 J/g. The extent of devitrification observed during small exothermic transitions could be manipulated by cooling and warming rates. Moderate to low cooling and warming rates are likely to result in thermal transitions (crystallization, recrystallization, melting) since these rates are too slow to outrun the kinetics of ice formation [41,42]. The DSC analysis in this study was done at cooling rates of 10 °C min^−1^, while plunging EAs into LN will significantly increase freezing rates [43]. The unfrozen water fraction was determined by using enthalpies as a function of water content [44,45]; the regression value obtained for *S. maire* EA was around 0.2 g/g. which is achieved by rapidly desiccating EAs for more than 210 min. For MC calculations and DSC analysis in this study, EAs were desiccated in batches and a single EA was used for DSC analysis while the rest of the EAs were used for MC determinations. This resulted in slight differences in total MC and the sum of osmotically active water content and osmotically inactive water content (Table 3) due to variation in embryo sizes.

In the absence of cryoprotection, it is critical that MC is lowered sufficiently to prevent the formation of large ice crystals that will be lethal to cells [43]. Ultrastructural observations of *S. maire* EAs at various steps in the desiccation and freezing process indicate that EAs with MCs > 0.75 g/g suffered complete disintegration of internal structures and major disruptions to the cell membranes. Rapid desiccation to MCs < 0.3 g/g resulted in plasmalemma irregularities, while frozen EAs with MCs < 0.3 g/g inspected in this study had intact cell membranes. It is, however, not clear whether the damage observed in this study was due to the desiccation or the subsequent fixation for transmission electron microscopy (TEM). Lack of resin infiltration when using standard fixation and embedding protocols has been reported for embryos of some species even when specimen size was dramatically reduced [46,47]. Ultrastructural observations of *S. maire* EAs also revealed various responses, from ultra-structurally intact to total lyses of the cells, even within the same treatment. Similar results have been reported for potato [48], *Cosmos atrosanguineus* [49], *Pisum sativum* [43] and Amaryllid species [50]. These authors highlighted that for an accurate assessment of the ultrastructural impacts associated with desiccation and cryopreservation, it is important that a large number of samples are assessed to determine the response of the explants to the treatment. Due to limited seed availability and access to electron microscopy, only five embryos per treatment were processed for ultrastructural studies of *S. maire* embryos. The images used in this study represented the general appearance of the cells for each treatment, but for a more accurate assessment of the ultrastructural response of *S. maire* to desiccation and freezing, a significantly larger sample size is required. 

## 4. Materials and Methods

### 4.1. Plant Material

Mature *S. maire* fruits were collected between December and March (2018–2021) from three sites in the North Island of New Zealand (39.2933° S, 174.2663° E) (Department of Conservation Permit number 69441-FLO) and one site in Nelson in the South Island. Fruits were transported to the Lions Ōtari Plant Conservation Laboratory in Wellington, where they were mixed with two parts medium = grade vermiculite (Ausperl, Australia) and stored in airtight containers at 5 °C until use. Fruits were not surface cleaned before storage. Prior to embryo excision, fruits were surface sterilized by immersion in 50% commercial bleach (4% sodium hypochlorite) for 5 min and then rinsed three times with sterile distilled water. The fruit pulp and seed coat were removed aseptically to expose the radicle tip, enabling excision of the embryonic axis.

### 4.2. Embryonic Axis Excision and In Vitro Germination for Viability Assessment

The embryonic axes (EAs) were excised by cutting through the cotyledon, leaving 1 mm of cotyledon still attached to the embryos. EAs were 4–5 mm in length. The EAs were sterilised by immersion in 5 g/L sodium dichloroisocyanurate (NaDCC) for 10 min, then rinsed three times using sterile distilled water. EAs were cultured on half-strength Murashige and Skoog [51] (MS) medium supplemented with 3% (*w*/*v*) sucrose and 0.2 g^−1^ ascorbic acid (AsA), hereafter referred to as growth medium (GM). Cryopreserved EAs were kept in the dark for one week prior to exposure to light. All EAs were grown at 15/25 °C with a 16 h light/8 h dark photoperiod provided by fluorescent tubes (PFD of 30–50 µmol/m²/s). EA survival was assessed using the criterion of normal seedling development, namely emergence of the radicle with geotropic growth of more than 5 mm (survival) and opening of the cotyledonary leaves (plantlet development). Embryo survival and plantlet development were assessed every 7 days. Survival was expressed as the percentage of final radicle emergence, while mean germination over time was used to assess the number of EAs that showed survival each week. 

### 4.3. Embryo Desiccation

EAs were excised in batches of 10. Prior to the desiccation treatment, EAs were submerged in 0.2 g^−1^ AsA, kept in the dark for a maximum of 15 min, blotted dry, and then desiccated. After the desiccation treatment, MCs of the EAs were determined gravimetrically after drying at 103 ± 1 °C for 17 h. Values represent the mean MC of 10 axes replicated four times and expressed as g H_2_O per g dry matter (g/g). To evaluate the impact of desiccation on embryo survival, the EAs were rehydrated for 30 min in the dark in one of four solutions: (a) sterile distilled water (DW), (b) 0.2 g^−1^ AsA, (c) calcium/magnesium solution (1 μM CaCl_2_ + 1 mM MgCl_2_) or (d) liquid MS. Rehydration was followed by sterilisation of the EAs and incubation in GM as described above. 

#### 4.3.1. Rapid Desiccation

Rapid desiccation was conducted in a modified chamber originally described by [1] (Supplementary Figure S2A). Plastic columns of 250 mm × 130 mm (height × diameter) were created using a ANet A8 3D printer. A computer cooling fan (12 V) was mounted in the middle of the column with airflow directed upwards towards the gauze, which supported the EAs. Two columns were placed in a 27 L airtight storage container containing 1 kg of activated silica gel (Supplementary Figure S2B). The air inlet into the pipe was buried in the silica gel to ensure uptake of desiccated air. EAs were desiccated in the chamber for 0, 60, 120, 150, 180 and 210 min.

#### 4.3.2. Slow Desiccation

EAs were placed on sterile filter paper and dried under laminar air current for 0, 1, 2, 3, 4, 5 or 6 h.

### 4.4. Cryopreservation of Desiccated EAs

Following the slow and rapid desiccation treatments described above, EAs were transferred to 1.8 mL sterile cryovials and plunged into liquid nitrogen (LN). After a minimum of 1 h in LN, EAs were rapidly thawed by stirring the cryovials in a water bath at 40 ± 2 °C for 2–3 min before rehydration in sterile 0.2 g^−1^ AsA for 30 min in the dark. Sterilized EAs were placed on GM and kept in the dark for at least 7 days. Survival and plantlet development were determined as described above.

### 4.5. Oxidative Stress

EAs were excised and rapidly desiccated for 0, 60, 120, 150, 180 and 210 min. After the desiccation treatment, EAs were rehydrated in 0.2 g^−1^ AsA or DW in the dark for 30 min. To reflect accumulated metabolic damage, EAs were recovered in the dark for 48 h in vitro and then stored in LN until further processing.

### 4.6. Extraction of Total Proteins and Semi-Purification of Protein Extracts

High levels of phenolics in the EAs were removed by adding polyvinylpolypyrrolidone (PVPP) to the extraction buffer and by the semi-purification of crude protein extracts by repeated ultrafiltration. These steps were essential to preserve enzyme activities. Frozen, pre-weighed EAs were placed in 2 mL screw-capped tubes with 900 μM ice-cold extraction buffer (EB; 100 mM potassium phosphate buffer (pH 7.0) containing 0.1 mM Na_2_ EDTA, 3% PVPP, and 1mM phenylmethylsulfonyl fluoride (PMSF). To homogenize the EAs, five 1.4 mm zirconia beads were added to each tube and the tubes were placed on ice. A mini-bead beater (Biospec mini^TM^) was used to homogenize the EAs for 10 sec at a time, repeated four times. 

Once the tissue was sufficiently homogenized, the tubes were centrifuged (Eppendorf Centrifuge 5424R, Germany) at 4 °C for 30 min at 15,000 rpm to obtain a pellet of cell debris and a supernatant containing the proteins (enzymes). The supernatant was then semi-purified to remove small interfering molecules by ultrafiltration using 0.5 mL Amicon^®^ Ultra centrifugal filters (10 kD cut-off). The supernatant (500 μL) was placed in the ultrafilter and centrifuged at 4 °C for 30 min at 10,000 rpm. The semi-purified protein was then reconstituted to 500 μL using 100 mM potassium phosphate buffer (pH 7.0) and the ultrafiltration process was repeated three times by reconstituting the supernatant with phosphate buffer and transferring the protein sample to a fresh ultrafilter. After the third ultrafiltration, the reconstituted protein sample was transferred to a 1.5 mL centrifuge tube and centrifuged for 5 min at 15,000 rpm. The semi-purified protein extracts were then stored at −80 °C until assayed, as detailed below. The protein contents of the semi-purified extracts were determined using a Lowry protein assay as per Fryer et al. [52] with a bovine serum albumin (BSA) standard. The pellet of cell debris was retained and used for the lipid extraction, as detailed below.

#### 4.6.1. Extraction of Lipids

The pellet retained from the protein extraction was homogenized in 0.6 mL methanol:chloroform (2:1 *v*/*v*) for 1 min before adding 0.4 mL chloroform and 0.4 mL deionised water and centrifuged for 30 s at 13,300 rpm. The phases were allowed to separate before transferring the chloroform phase to new tubes. All samples were stored at –80 °C until analysed.

#### 4.6.2. Antioxidant Enzyme Assays

Superoxide dismutase (SOD) activity was assayed using the semi-purified protein extracts following the microplate assay protocol of [53] with minor modifications. The activity in each of the extracts was calculated with reference to a standard (prepared from bovine liver SOD (Sigma-Aldrich, St. Louis, MO, USA)) and expressed as units of SOD mg^−1^ of total protein. One unit of SOD corresponds to the amount of enzyme that inhibits the reduction of cytochrome c by 50% in a coupled system with xanthine oxidase at pH 7.8 and 25 °C.

Catalase (CAT) was assayed using the methodology developed by [54] and adapted by [55] for 96-well microplates. The activity in each of the extracts was calculated with reference to a standard (purified bovine liver CAT; Sigma-Aldrich, St. Louis, MO, USA) and expressed as μM of H_2_O_2_ consumed min^−1^ mg^−1^ of total protein.

The glutathione peroxidase (GPOX) activity was assayed using the spectrophotometric method described by [56], with modifications for use with a microplate reader. GPOX activity was calculated with reference to a standard (GPOX purified from bovine erythrocytes; Sigma-Aldrich, St. Louis, MO, USA) and expressed as nmol min^−1^ mg^−1^ of total protein.

#### 4.6.3. Protein Carbonyls

Protein carbonyl (PC) levels were determined in the semi-purified protein extracts via reaction with 2.4-dinitrophenylhydrazine (DNPH) [57], adapted for measurement in a microplate reader. Protein carbonyl contents (nmol) were determined using the extinction coefficient of DNPH at 370 nm (0.022 μM^−1^ cm^−1^), corrected for the calculated pathlength of the solution (0.6 cm).

#### 4.6.4. Lipid Peroxide Assay

The lipid peroxide (LPOx) content was determined using the ferric thiocyanate [58] method adapted for measurement in a microplate reader using glass microplates. A calibration curve with t-butyl hydroperoxide was used and lipid peroxide content was calculated as nmol lipid peroxide g/g FW. 

### 4.7. Differential Scanning Calorimetry (DSC)

Thermal behaviour in the EAs for each desiccation period (0, 60, 120, 150, 180 and 210 min) was determined using a Perkin-Elmer differential scanning calorimeter (DSC 8500) (Shelton, CT, USA), calibrated for temperature and heat flow with zinc (melting point 419.5 °C) and indium (melting point 156.6 °C). One EA per treatment was hermetically sealed into a large volume (60 µL) stainless steel capsule (sample pan) using an O-ring with the aid of a Perkin-Elmer Universal Crimper (Shelton, USA). Sample weight was measured using a micro-balance (Model: XPR6UD5; Mettler-Toledo, Switzerland), and subjected to calorimetric assessment within 5 min of sample preparation. Samples were cooled from 25 °C to −100 °C and held for 1 min before rewarming to 25 °C at a rate of 10 °C min^−1^. Exothermic- and endothermic-heat changes were derived from the crystallization and melt endotherm during the cooling and warming cycles. The onset temperature of the transition was determined as the temperature at which the tangent of the sharpest portion of the first peak intersected the baseline. The onset, peak and end temperatures were calculated using PYRIS software. The areas of melt and crystallization peaks were calculated from the area above the baseline and expressed as millijoule (mJ). The enthalpies for these transitions are presented as Joule per gram of sample weight (J/g). Calorimetric data were collected from three replicates per treatment. After calculating the dry weight of each sample (as described above), the frozen and unfrozen water proportions were calculated. Since 1 g of water releases 334.5 joules of heat energy during the conversion to and from ice, the osmotically active water content of the sample was calculated from the endothermic heat changes derived from the melting endotherm [59,60].

### 4.8. Ultrastructural Observations—Transmission Electron Microscopy (TEM)

The effects of desiccation stress are unlikely to be instantaneous; thus, freshly excised EAs and those subjected to desiccation with and without freezing, were processed for TEM after rehydration and a subsequent 48 h in the in vitro recovery period. To study the effect of dehydration and freezing on the ultrastructure of meristematic cells in EAs, photomicrographs of desiccated root meristems were compared between fresh, desiccated and desiccation plus frozen samples. Immediately after the desiccation treatment (0, 60, 120, 150 or 180 min), EAs were placed in 2 mL cryovials and stored in LN for 24 h. After 2 min thawing at 40 °C, EAs were rehydrated in 0.2 g^−1^ AsA for 30 min. Non-frozen samples were rehydrated after the desiccation treatment. EAs were then recovered on GM for 48 h in the dark before small blocks containing the root meristems were fixed for TEM. Samples were fixed in 2.5% phosphate-buffered glutaraldehyde (0.1 M, pH 7.2) for 24 h at 4 °C. After several rinses with phosphate buffer, specimens were post-fixed in 0.5% aqueous osmium tetroxide for 1 h at room temperature and rinsed three times with phosphate buffer. Specimens were then dehydrated in a graded acetone series (30%, 50%, 75% for 5 min), followed by 100% acetone for 10 min. This was followed by infiltration and embedding in low-viscosity resin and polymerisation for 8 h at 70 °C. Specimens were sectioned using Ultracut E ultramicrotome (Leica, Austria). Ultra-thin sections of root meristem showing copper/gold interference colours were collected on 600 mesh copper grids and contrasted for electron microscopy using a standard double-staining procedure saturated (2.5%) uranyl acetate followed by lead citrate. TEM was carried out at the Manawatu Microscopy and Imaging Centre, Massey University, Palmerston North.

### 4.9. Statistical Analysis

All germination experiments involved 10 EAs for each treatment, repeated four times. Survival and plantlet assessment results were compared using ANOVA, followed by Fisher’s protected least significant difference test for significantly different means (at *p* < 0.05). Inter-treatment differences in lipid peroxidation, protein carbonyls and enzyme activity were tested using ANOVA, followed by Tukey’s HSD test for pairwise comparisons. Correlations between viability and antioxidant enzyme activity were tested using Pearson correlation analysis. For correlation analysis, percentages were transformed (arcsin) to conform to parametric test assumptions. ANOVA followed by Tukey’s HSD test was used to investigate differences in onset of melt temperature, freeze temperature and enthalpy of the melt. All statistical analysis was performed at 0.05 level of significance, and results are expressed as mean ± SD. Statistical analysis was conducted using XLStat Software version 1.3 (2021) and SAS/STAT version 14.2.

## 5. Conclusions

This study investigated the reasons for desiccation sensitivity in *S. maire* EAs and whether the formation of intracellular ice causes freezing damage in EAs desiccated to various MC levels. The decline in enzymatic antioxidant activities and the increase in lipid peroxidation suggest that oxidative stress, rather than structural damage, causes viability loss in EAs desiccated to MCs < 0.3 g/g. This was supported by thermal analysis, which showed that endothermic peaks are significantly reduced at lower MCs with MCs of <0.2 g/g required to eliminate intracellular ice formation. However, this level of desiccation was found to be lethal to EAs. As cryopreservation is the only viable option to conserve *S. maire*, future perspectives for successful cryopreservation of this species should investigate faster cooling and warming rates coupled with the exogenous application of antioxidants to increase EA survival following desiccation for embryo cryopreservation. In addition, rapid desiccation in combination with cryoprotective agents such as Plant Vitrification Solutions 2 or 3 (PVS2 or PVS3), and the addition of antioxidants is recommended to promote embryo survival following freezing. Embryo plumule cryopreservation could also be another potential technique for the conservation of this species. 

## Figures and Tables

**Table 1:** Comparison of embryo moisture content (MC) and survival (%) before (-LN) and after (+LN) liquid nitrogen cryopreservation for embryonic axes desiccated slowly (0–6 h) or rapidly (0–210 min). Results are displayed as mean ± SD. Survival values for each desiccation method followed by the same letter do not differ significantly (Fisher’s, *p* < 0.05; N = 10).

Slow Desiccation			
Desiccation Time (min)	Embryo MC (g/g)	Survival (%)	
		−LN	+LN
0	1.860 ± 0.51	100 ± 0.0 ^a^	0
60	1.019 ± 0.25	100 ± 0.0 ^a^	0
120	0.731 ± 0.25	100 ± 0.0 ^a^	0
180	0.457 ± 0.18	86.6 ± 15.3 ^ab^	0
240	0.643 ± 0.31	40 ± 17.3 ^def^	0
300	0.478 ± 0.06	16.7 ± 11.5 ^fg^	0
360	0.407 ± 0.18	0 ^g^	0
**Rapid Desiccation**			
**Desiccation Time** **(min)**	**Embryo MC** **(g/g)**	**Survival (%)**	
		**−LN**	**+LN**
0	1.94 ± 0.93	100 ± 0 ^a^	0
60	0.75 ± 0.06	78.7 ± 30.8 ^ab^	0
120	0.44 ± 0.15	56.5 ± 24.6 ^b^	0
150	0.35 ± 0.06	50.7 ± 20.1 ^bc^	0
180	0.28 ± 0.05	28.5 ± 30.2 ^cd^	0
210	0.2 ± 0.02	0 ^d^	0

**Table 2:** The effect of rapid desiccation and rehydration solution on embryo germination, SOD, CAT, GPOX activities and PC and LPOx levels. Data are presented as mean ± SD (*p* < 0.05) for six different treatments. Superscript of different letters within a column indicates significant differences at *p* < 0.05 based on a one-way ANOVA analysis (Tukey HSD test).

Dry Time (min)	MC (g/g)	Rehydration	Embryo Survival (%)	Enzymatic Antioxidants			Damage Markers	
				SOD (U mg^−1^ prot)	CAT (nmol/min/mg prot)	GPOX (nmol/min/mg prot)	PC (nmol/mg prot)	LPOx (nmol/g/FW)
0	1.94 ± 0.2	n/a	100 ± 0.0	67.6 ± 8.1 ^a^	46.9 ± 4.96 ^a^	49.19 ± 3.06 ^a^	5.38 ± 0.85 ^a^	7.00 ± 0.82 ^a^
60	0.75 ± 0.12	AsA	100 ± 0.0	63.9 ± 11.5 ^ab^	45.5 ± 6.5 ^ab^	49.5 ± 4.9 ^a^	6.0 ± 0.6 ^ab^	6.3 ± 0.3 ^a^
		DW	100 ± 0.0	60.5 ± 10.5 ^ab^	44.6 ± 5.4 ^ab^	44.6 ± 5.0 ^ab^	6.2 ± 0.2 ^ab^	5.9 ± 0.1 ^a^
120	0.45 ± 0.17	AsA	87.5 ± 15	49.2 ± 4.6 ^b^	38.5 ± 4.5 ^ab^	37.4 ± 1.5 ^bc^	7.5 ± 1.2 ^ab^	9.7 ± 1.4 ^a^
		DW	53.0 ± 24.5	49.1 ± 7.3 ^b^	35.7 ± 0.2 ^bc^	33.1 ± 4.8 ^cd^	7.3 ± 0.7 ^ab^	9.4 ± 1.7 ^a^
150	0.30 ± 0.01	AsA	37.0 ± 17.8	5.1 ± 0.6 ^d^	3.9 ± 0.2 ^d^	3.9 ± 0.8 ^e^	28.8 ± 5.3 ^c^	39.6 ± 3.2 ^b^
		DW	n/a	5.4 ± 0.3 ^d^	3.9 ± 0.4 ^d^	3.3 ± 0.3 ^e^	29.1 ± 2.9 ^c^	32.0 ± 2.7 ^b^
180	0.28 ± 0.07	AsA	49.3 ± 17.2	28.1 ± 1.9 ^c^	26.9 ± 1.8 ^c^	28.0 ± 1.1 ^d^	14.6 ± 1.6 ^b^	27.9 ± 5.3 ^b^
		DW	81.7 ± 18.3	ND	ND	ND	31.3 ± 3.5 ^c^	68.9 ± 3.0 ^c^
210	0.20 ± 0.03	AsA	0	ND	ND	ND	30.3 ± 2.8 ^c^	67.9 ± 13.3 ^c^
		DW	0	ND	ND	ND	29.9 ± 6.5 ^c^	74.7 ± 4.7 ^c^
F-value				69.39	98.63	162.5	43.6	95.98

AsA = Ascorbic acid, DW = Distilled water, ND = Not detected, SOD = Superoxide dismutase, CAT = Catalase, GPOX = Glutathione peroxidase, PC = Protein carbonyls, LPOx = Lipid peroxides.

**Table 3:** The effect of desiccation time (0–210 min), embryo weight (mg), total moisture content (MC) and water content (frozen and unfrozen water) of *Syzygium maire* embryonic axes (EAs).

Dry Time (min)	Embryo Weight (mg)	Total MC (g/g)	Water Composition	
			* Osmotically Active Water Content (Frozen Water) (g/g)	** Osmotically Inactive Water Content (Unfrozen Water) (g/g)
0	5.21 ± 0.6	1.94 ± 0.2	2.36 ± 1.0	0.38 ± 0.05
60	2.96 ± 0.8	0.75 ± 0.12	0.71 ± 0.2	0.18 ± 0.05
120	2.74 ± 0.4	0.45 ± 0.17	0.38 ± 0.07	0.04 ± 0.03
150	2.13 ± 0.1	0.30 ± 0.01	0.33 ± 0.1	0.04 ± 0.03
180	3.20 ± 0.5	0.28 ± 0.07	0.21 ± 0.01	0.06 ± 0.01
210		0.20 ± 0.03	ND	ND

ND = Not Detected. * Osmotically active water content of each EA was calculated from the endothermic-heat changes derived from the melt endotherm during the warming cycle, and the total water content of the sample as 1 g of water releases 334.5 joules of heat energy during the conversion to and from ice. ** Osmotically inactive water content was the difference between total water and osmotically active water contents.

## Data Availability

The data presented in this study are available on request from the corresponding author. The data are not publicly available due to the privacy statement in the original project.

## Supplementary Materials

The following supporting information can be downloaded at: https://www.mdpi.com/article/10.3390/plants11081056/s1, Figure S1: The impact of desiccation on *Syzygium maire* plantlet regeneration; Figure S2: Rapid desiccation chamber. Table S1: Thermodynamic properties of *Syzygium maire* embryonic axes (EAs) following rapid desiccation.
