# Supplementary material for: Impacts of Rapid Desiccation on Oxidative Status, Ultrastructure and Physiological Functions of Syzygium maire (Myrtaceae) Zygotic Embryos in Preparation for Cryopreservation
Source: Plants (Basel). 2022 Apr 13;11(8):1056. doi: 10.3390/plants11081056 (PMC9028110; doi:10.3390/plants11081056)
Supplement: Supplementary file 1 [file plants-11-01056-s001.zip › plants-1652562-supplementary.pdf]

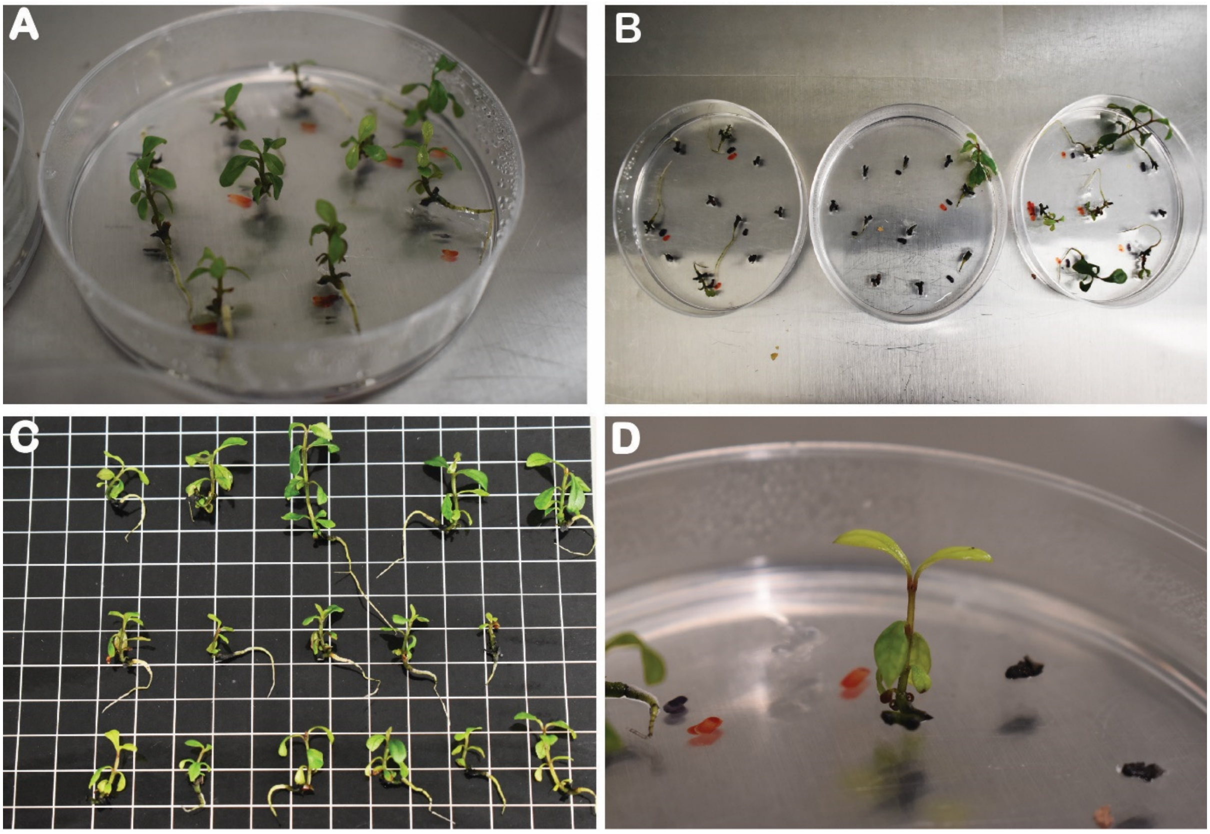

Figure S1. The impact of desiccation on Syzygium maire plantlet regeneration. Compared to untreated embryonic axes (EAs) (**A**), desiccation to moisture content below 0.28 g/g significantly reduced the regeneration of plantlets (**B**). After 4 months *in vitro* plantlets from all treatments were of similar size (**C**) although a few plantlets produced shoots but no root following desiccation (**D**).

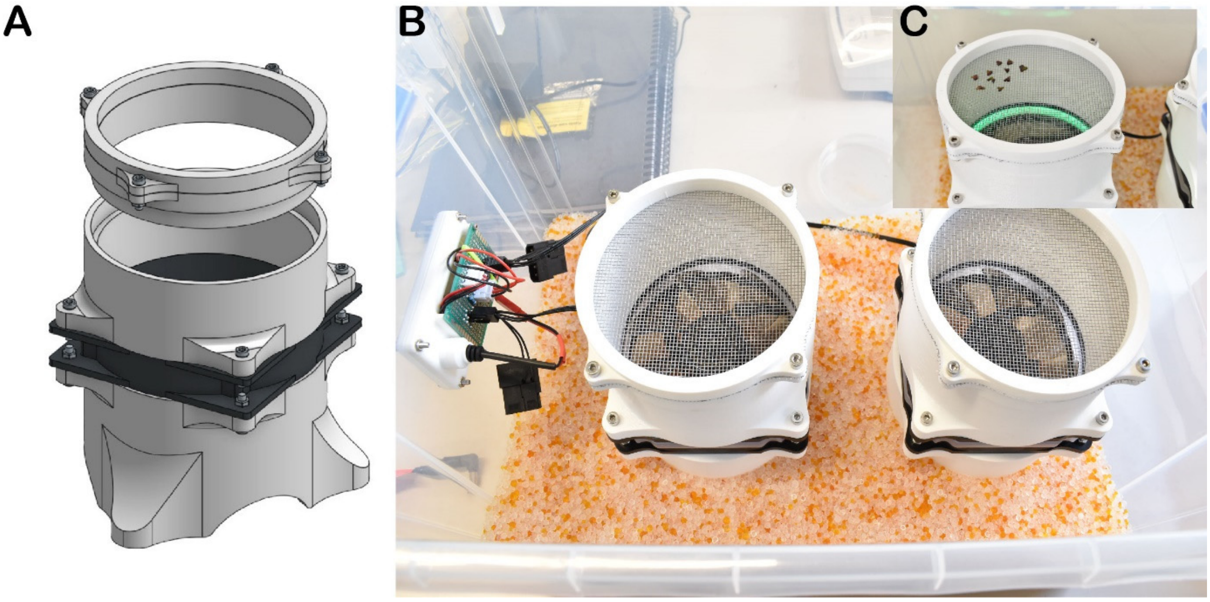

Figure S2: Rapid desiccation chamber. Rapid desiccation chamber showing location of gauze (**A**) in the 3D printed plastic column with two columns (**B**) located in an airtight container with silica gel. Insert (**C**) illustrates *Syzygium maire* EAs on the gauze.

Table S1. Thermodynamic properties of Syzygium maire embryonic axes (EAs) following rapid desiccation. Desiccation time (0–210 min) as well as cooling and warming thermodynamic properties of *Syzygium maire* embryonic axes (EAs). Superscript of different letters within a column indicates significantly difference at $p < 0.05$ based on a one-way ANOVA analysis (Tukey HSD test). Data are presented as mean ± SD ($P < 0.05$) for three different treatments.

Dry Time (min)	Cooling				Warming			
	**Onset Temperature of Crystallization (°C)**	**End Temperature of Crystallization (°C)**	**Area of crystallization (mJ)**	**Enthalpy of Crystallization (J/g)**	**Onset Temperature of Melt (°C)**	**End Temperature of Melt (°C)**	**Area of Melt (mJ)**	**Enthalpy of Melt (J/g)**
0	–3.05 ± 3.1a	–22.8 ± 1.0a	–571.0 ± 189.9a	109.5 ± 20.2a	9.3 ± 2.2a	21.8 ± 3.0a	603.6 ± 122.2a	128.0 ± 16.3a
60	–4.21 ± 6.8a	–23.6 ± 3.5a	–213.8 ± 86.6b	70.1 ± 10.6ab	8.3 ± 4.4a	19.6 ± 2.4ab	180.5 ± 87.2b	60.4 ± 18.0b
120	–18.74 ± 2.7a	–26.0 ± 1.9a	–74.5 ± 36.2b	27.5 ± 13.0bc	0.9 ±1.3b	16.4 ± 1.3b	35.2 ± 32.0b	13.9 ± 10.0c
150	–12.85 ± 10.0a	–25.7 ± 1.7a	–49.7 ± 23.7b	23.7 ± 12.5bc	–1.3 ± 0.5b	16.0 ± 1.0b	28.4 ± 17.8b	13.3 ± 9.2c
180	–13.5 ± 4.6a	–28.6 ± 1.4a	–80.9± 52.6b	24.3 ± 12.7bc	–2.0 ± 2.6b	16.5 ± 0.7b	68.5 ± 21.7b	20.3 ± 4.4c
210	–7.77 ± 0a	–30.3 ± 0a	–12.8 ± 0b	7.0 ± 0c	ND	ND	ND	ND
